# Serotonin as a Regulator of Innate and Adaptive Immunity: Mechanisms and Clinical Relevance in Inflammatory Disease

**DOI:** 10.3390/cells15151353

**Published:** 2026-07-28

**Authors:** Vladimir Inozemtsev, Vera Shashkovskaya, Viktoria Sergunova

**Affiliations:** Federal Research and Clinical Center of Intensive Care Medicine and Rehabilitology, V.A. Negovsky Research Institute of General Reanimatology, 107031 Moscow, Russia; verashashkovskaya@googlemail.com

**Keywords:** peripheral serotonin, 5-hydroxytryptamine, immune regulation, innate immunity, adaptive immunity, inflammation, serotonergic signaling, macrophage polarization, NETosis, inflammatory diseases

## Abstract

Serotonin (5-hydroxytryptamine, 5-HT) is traditionally considered a neurotransmitter, but most of the body’s serotonin is peripheral and participates in vascular, hemostatic, inflammatory, and immune processes. This review summarizes current evidence on the role of peripheral serotonin in innate and adaptive immunity and evaluates its clinical relevance in inflammatory and immune-mediated diseases. The article synthesizes experimental and clinical data on serotonin sources, 5-HT receptors, SERT, serotonylation, and 5-HT-dependent regulation of neutrophils, monocytes/macrophages, mast cells, dendritic cells, T cells, and B cells. The reviewed data indicate that serotonin modulates leukocyte recruitment, platelet–immune interactions, NETosis, macrophage polarization, dendritic cell–T-cell communication, lymphocyte activation, vascular permeability, and tissue remodeling. Clinically, serotonergic mechanisms are implicated in sepsis, inflammatory bowel disease, autoimmune disorders, cardiovascular inflammation, thromboinflammation, and the tumor microenvironment. However, the direction of 5-HT effects is not uniform and depends on receptor subtype, cell type, local mediator concentration, disease stage, and tissue context. Serotonin should therefore be regarded as a context-dependent immunoregulatory mediator rather than an exclusively pro- or anti-inflammatory factor. Further studies are needed to standardize clinical measurements of serotonin and its metabolites and to define therapeutically relevant serotonergic targets.

## 1. Introduction

Serotonin (5-hydroxytryptamine, 5-HT) is traditionally described as a neurotransmitter of the central nervous system that regulates mood, behavior, sleep, appetite, and neuroendocrine processes [1]. This view is incomplete because most serotonin in the body is located outside the central nervous system. Peripheral 5-HT is synthesized mainly by intestinal enterochromaffin cells, taken up by platelets, and released during platelet activation. It contributes to vascular tone, hemostasis, and inflammatory reactions [2,3].

Immunological and pharmacological studies describe serotonin as an immunoregulatory mediator. Components of the serotonergic system, including 5-HT receptors, the serotonin transporter SERT, and enzymatic pathways of 5-HT metabolism, have been detected in different immune cell populations [3,4]. Through these mechanisms, serotonin can modify leukocyte migration, cytokine secretion, antigen presentation, lymphocyte activation, intercellular communication, and vascular inflammatory interactions [3,4,5]. The effects of 5-HT are not unidirectional and depend on the cell type, receptor profile, local mediator concentration, and type of pathological process [3,4,6].

Data on this topic are distributed across neurobiological, gastroenterological, pharmacological, and immunological studies. Immunological aspects are often discussed separately from the clinical context [2,3,4]. The results of individual studies are also difficult to compare because peripheral and central serotonin differ, methods for measuring 5-HT in blood are not uniform, and the effects of 5-HT are receptor- and tissue-specific [2,7].

This review is structured around three related areas. The first section addresses innate immunity because cells of the early inflammatory response, including neutrophils, monocytes, macrophages, dendritic cells, and platelet–leukocyte complexes, are among the first cells to interact with peripheral serotonin in blood and tissues [3,5,8]. The second section addresses adaptive immunity because 5-HT can affect T-cell activation, B-cell function, immune tolerance, and intercellular signaling through antigen-presenting cells [4,9]. The third section considers the clinical role of serotonin in diseases associated with inflammation and immune dysregulation, including sepsis, inflammatory bowel disease, autoimmune pathology, cardiovascular inflammation, and the tumor microenvironment [7,10,11,12].

The structure of the review links cellular mechanisms of 5-HT action with clinical phenotypes and therapeutic targets. Pharmacological modulation of the serotonergic system through SERT, TPH1, and specific HTRs is also discussed in the context of inflammatory diseases, sepsis, and antitumor therapy [12,13,14].

The aim of this review is to summarize current knowledge on the role of serotonin in the regulation of innate and adaptive immunity and to assess the clinical relevance of serotonergic mechanisms in inflammatory and immune-mediated disorders.

## 2. Serotonin and Innate Immunity

### 2.1. Sources of Peripheral Serotonin in the Immune Response

Peripheral serotonin is functionally separated from central serotonin because 5-HT does not cross the blood–brain barrier in physiologically significant amounts. The main source of peripheral serotonin is intestinal enterochromaffin cells, where 5-HT synthesis depends mainly on TPH1 [2,7]. After release from enterochromaffin cells, serotonin can enter the bloodstream and be taken up by platelets through the serotonin transporter SERT/SLC6A4 [3,7]. Platelets are the main circulating reservoir of 5-HT because the absorbed serotonin accumulates in dense granules through VMAT2 and is released during platelet activation at sites of vascular injury, inflammation, or thrombosis [3,4,7].

This separation should be interpreted as a compartmental distinction rather than only as an anatomical statement. Circulating 5-HT is largely controlled by intestinal synthesis, platelet uptake through SERT, peripheral storage, and peripheral metabolism, whereas central 5-HT is synthesized mainly in serotonergic neurons. Therefore, peripheral 5-HT measurements should not be used as a direct surrogate for central serotonergic activity, even when inflammatory or neuroimmune outcomes are discussed [2,7].

Mast cells are also considered a tissue source of serotonin, especially in allergic and neurogenic inflammation. Serotonin is one of the preformed mediators that can be stored in mast cell secretory granules and released after activation [15]. Local tissue pools of serotonin are relevant to the paracrine regulation of immune cells because 5-HT concentration in the inflammatory microenvironment can differ from that in plasma [15,16]. Immune cells can respond to serotonin not only through receptors but also by participating in its uptake, transport, or local transfer, as shown for dendritic cells and the lymphocytic immunological synapse [17].

### 2.2. 5-HT Receptors and SERT in Innate Immune Cells

Serotonin mediates cellular effects through several families of receptors, including 5-HT_1_, 5-HT_2_, 5-HT_3_, 5-HT_4_, 5-HT_5_, 5-HT_6_, and 5-HT_7_. Most 5-HT receptors are G protein-coupled; the exception is the 5-HT_3_ receptor, a ligand-gated ion channel [18]. Immune cells express various components of the serotonergic system (Table 1), including different 5-HT receptors, SERT/SLC6A4, and enzymes involved in serotonin metabolism or intracellular serotonin effects [4,5,16].

In innate immune cells, serotonergic effects depend on the cell type and receptor profile [5,16]. In monocytes and macrophages, 5-HT can change cytokine production and the transcriptional profile of the inflammatory response [5,22]. In dendritic cells, serotonin affects maturation, migration, and the ability to activate T cells [17,26]. In neutrophils, serotonin is mainly associated with regulation of recruitment, interactions with the vascular wall and platelets, and NETosis [8,19]. SERT mediates cellular uptake of 5-HT and affects both the extracellular concentration of serotonin and the possibility of intracellular 5-HT effects [7,17].

In addition to receptor-dependent mechanisms, serotonin can participate in covalent post-translational protein modification, termed serotonylation. Serotonylation is mediated by transglutaminases and involves the attachment of serotonin to glutamine residues in proteins [46]. This process has been described in intracellular signaling, platelet activation, and transcriptional regulation through histone serotonylation. In the immune system, the role of serotonylation has not been studied uniformly: the molecular mechanism is established, but the cell-specific roles across different innate immune populations require additional experimental verification [46,47].

The functional effects of serotonin in immune and inflammation-associated cells cannot be explained by receptor expression alone. They also depend on downstream signaling, cellular uptake through SERT, vesicular storage, peripheral synthesis, and receptor-independent intracellular mechanisms such as serotonylation. Table 2 summarizes the principal receptor-dependent and non-receptor serotonergic pathways discussed throughout the following sections and illustrates how differences in signaling and cellular context may result in inflammatory, regulatory, profibrotic, or antitumor effects.

### 2.3. Neutrophils

Neutrophils are innate immune cells involved in the early phase of inflammation, phagocytosis, reactive oxygen species production, degranulation, and formation of neutrophil extracellular traps [20]. Serotonin affects the neutrophil response mainly through a platelet–vascular context because activated platelets release 5-HT and support leukocyte adhesion and recruitment to inflammatory sites [8].

In an experimental model of acute inflammation in mice, platelet serotonin increased neutrophil recruitment into inflamed tissues, whereas the absence of platelet serotonin was associated with reduced neutrophil infiltration and improved survival during LPS-induced endotoxic shock [8].

Serotonin can contribute to the regulation of neutrophil–endothelial interactions by modulating the vascular wall and platelet activation. Platelets support the early stages of neutrophil recruitment, including tethering, rolling, arrest, and firm adhesion, which links serotonin to cellular processes of neutrophil extravasation [8,49].

Platelet–neutrophil aggregates participate in neutrophil activation and NET formation. NETosis is a specialized neutrophil response characterized by chromatin decondensation, loss of nuclear segmentation, cell spreading, and the subsequent release of neutrophil extracellular traps (NETs), which consist mainly of DNA, histones, and granular antimicrobial proteins [48,54,55]. Although NETs contribute to extracellular pathogen immobilization and elimination, excessive or dysregulated NET formation may promote inflammation, tissue injury, and thromboinflammatory complications [56,57]. NETs can promote thrombosis because extracellular DNA, histones, and neutrophil granular proteins contribute to activation of coagulation and platelets [20,49,58]. In sepsis-induced lung injury, platelet-derived 5-HT was associated with increased NET formation, suggesting a possible role for serotonin in immunothrombosis and organ damage [19,53].

Direct receptor-mediated regulation of the neutrophil respiratory burst by 5-HT remains poorly characterized. The available primary evidence instead supports the involvement of serotonin in platelet-dependent neutrophil recruitment, platelet–neutrophil interactions, and NET formation. Therefore, these effects should not be interpreted as evidence that 5-HT consistently activates or inhibits NOX2-dependent reactive oxygen species production in neutrophils [8,21,33].

Liu et al. described a possible direct mechanism by which 5-HT participates in NETosis: serotonin was taken up by neutrophils through SERT and induced TGM2-dependent histone serotonylation coupled with PAD4-dependent citrullination. These processes facilitated chromatin decondensation and NET formation. Blocking 5-HT uptake with fluoxetine or suppressing TGM2 reduced H3Q5ser/H3cit modifications and decreased NET formation; current support for this pathway is strongest in models of neuroendocrine tumors and liver metastasis [21].

### 2.4. Monocytes and Macrophages

Monocytes and macrophages express components of the serotonergic system and respond to 5-HT by changing cytokine, chemokine, and transcriptional profiles. Serotonin regulates production of TNF-alpha, IL-1beta, IL-6, IL-10, and several chemokines, but the direction of the effect depends on receptor subtype, stage of cell differentiation, type of inflammatory stimulus, and tissue microenvironment [5,21,22].

TNF-alpha, IL-1beta, and IL-6 amplify inflammation by promoting endothelial activation, leukocyte recruitment, fever, and the acute-phase response. IL-10 limits macrophage and T-cell activation, whereas chemokines such as CCL2/MCP-1 guide leukocyte migration into inflamed tissues [59,60].

In human monocyte-derived macrophages, 5-HT shifted the macrophage phenotype toward an M2-like profile mainly through 5-HT_2_B and 5-HT_7_. In this model, serotonin reduced LPS-induced production of pro-inflammatory mediators and changed the expression of genes related to anti-inflammatory regulation, tissue remodeling, and tissue repair [22].

Later data showed that the 5-HT_7_ receptor-dependent action of 5-HT is mediated through the 5-HT_7_-cAMP/PKA signaling axis and is associated with an anti-inflammatory and profibrotic transcriptional profile, including increased TGF-beta1 production. In a mouse model of skin fibrosis, absence of the 5-HT_7_ receptor was associated with reduced macrophage infiltration and collagen deposition, supporting a role of this receptor in fibrotic remodeling in vivo [23]. TGF-beta1 has both immunoregulatory and profibrotic functions and promotes extracellular-matrix deposition during chronic tissue remodeling [23].

5-HT_2_B-dependent effects of 5-HT include a distinct molecular component: in human macrophages, serotonin activated the 5-HT_2_B-AhR axis and altered the expression of aryl hydrocarbon receptor target genes involved in immune regulation and xenobiotic responses [24]. 5-HT_7_ has also been shown to affect cytokine secretion, morphology, phagocytic activity, and migratory activity of macrophages; the magnitude of the effect depends on whether the initial state is M0-, M1-, or M2-like [9].

A separate form of serotonergic regulation has been described in alveolar macrophages. In murine alveolar macrophages, 5-HT acted through 5-HT_2_C, increased intracellular Ca^2+^, and enhanced CCL2/MCP-1 production [25].

M0 macrophages are generally considered non-polarized or baseline-differentiated cells, whereas M1-like macrophages represent a pro-inflammatory state induced by IFN-gamma and microbial signals such as LPS and characterized by production of TNF-alpha, IL-1beta, IL-6, IL-12, reactive oxygen/nitrogen species, and chemokines involved in leukocyte recruitment. By contrast, M2-like macrophages are associated with IL-4/IL-13-, IL-10-, glucocorticoid-, or tissue repair-related signals and are characterized by enhanced efferocytosis, matrix remodeling, angiogenesis, anti-inflammatory mediator production, and expression of markers such as CD206, CD163, ARG1, MRC1, IL-10, TGF-beta, and CCL18, although these markers vary between human and murine macrophages and across tissues [59,61,62]. Morphologically, M1-like macrophages are often described as more rounded or flattened cells, whereas M2-like macrophages more frequently acquire an elongated, spindle-like, or branched morphology; however, these differences are context-dependent and should not be used as independent markers of polarization [51,63,64].

The M1/M2 classification describes only extreme states of macrophage activation. A shift toward M2 induced by 5-HT should not be interpreted as universal suppression of inflammation. Serotonin can simultaneously limit production of pro-inflammatory cytokines and support tissue remodeling, fibrosis, and changes in macrophage phagocytic or migratory activity [22,60,65].

This distinction explains part of the apparently opposing macrophage effects of 5-HT. In human monocyte-derived macrophages, 5-HT reduced LPS-induced pro-inflammatory mediators and shifted cells toward an M2-like program through 5-HT_2_B- and 5-HT_7_-related mechanisms. At the same time, 5-HT_7_ receptor–cAMP/PKA signaling was associated with a profibrotic transcriptional program and increased TGF-beta1 production, while 5-HT_2_B signaling activated the AhR axis and changed immune-regulatory and xenobiotic-response genes. These findings show that anti-inflammatory cytokine modulation and tissue-remodeling or profibrotic activity may occur simultaneously rather than represent mutually exclusive outcomes [22,23,24].

### 2.5. Mast Cells, Eosinophils, and Basophils

Mast cells are tissue-resident innate immune cells involved in allergic inflammation, anaphylaxis, protection of barrier tissues, and neuroimmune interactions. Serotonin is part of the mediator spectrum associated with mast cells and can be released after their activation. In tissues, mast cell-derived serotonin can participate in regulation of vascular permeability, smooth muscle activity, and neuroimmune signaling [15,30]. In an experimental model of inflammatory pain, mast cell-derived serotonin contributed to neuroimmune mechanisms of pain sensitivity, but this finding should not be generalized to all types of inflammation [31].

Eosinophils are involved in allergic inflammation, antiparasitic responses, and mucosal inflammation. Serotonergic signaling through 5-HT_2_ receptors has been linked to regulation of airway inflammation in experimental systems. In a mouse model of allergic asthma, activation of 5-HT_2_ receptors by DOI reduced airway hyperresponsiveness, mucus production, inflammatory infiltration, and eosinophil recruitment. This finding indicates that 5-HT_2_-dependent effects in allergic inflammation may be anti-inflammatory in some models, although serotonin can enhance inflammatory reactions in other settings [16,24].

## 3. Serotonin and Adaptive Immunity

### 3.1. T Lymphocytes

T lymphocytes express several components of the serotonergic system, including specific 5-HT receptor subtypes and the serotonin transporter SERT/SLC6A4. Receptor expression depends on the activation stage, lymphocyte subpopulation, and microenvironmental conditions. Activated T cells show increased sensitivity to serotonergic signals, indicating the involvement of 5-HT in regulating the immune response after antigen stimulation [5,9].

Serotonin can act as a co-stimulatory signal during T-cell activation. In an anti-CD3/CD28 stimulation model, 5-HT increased early T-cell activation and the proliferative response via the 5-HT_7_ receptor; blockade of this receptor reduced the effect. Activated T cells can also synthesize serotonin de novo, allowing autocrine or paracrine regulation within lymphoid tissue [9].

Serotonin affects the T-lymphocyte cell cycle and can support the survival of proliferating cells. In several models, 5-HT increased the viability of mitogen-activated T cells, reduced apoptotic signals, and enhanced proliferation. These effects depended on the cell’s receptor profile and serotonin concentration [28].

The effect of serotonin on the Th1/Th2/Th17/Treg balance is context-dependent. In autoimmune models, 5-HT signaling has been linked to enhanced Th17 responses and inflammatory pathology. In other systems, increased IL-10 production and regulatory mechanisms associated with the Treg response have been described. A universal direction of 5-HT effects on T-helper differentiation has not been established [3,12].

Primary studies illustrate this context dependence: 5-HT_7_ signaling enhanced early T-cell activation in an anti-CD3/CD28 model [9], serotonin promoted Th17-associated pathology in experimental autoimmune arthritis [27], whereas 5-HT_7_ activation increased IL-10 production in PBMCs from natalizumab-treated patients with multiple sclerosis [49].

5-HT_7_ is one of the most studied serotonin receptors in T cells. Its activation is associated with increased intracellular cAMP, cytoskeletal changes, migration, and enhanced early activation of T lymphocytes [9,29]. In some inflammatory diseases, HTR7 expression on T cells is altered, which makes it a potential biomarker or therapeutic target [29].

### 3.2. B Lymphocytes

B lymphocytes express serotonin receptors and can respond to 5-HT by changing survival and proliferation [28,66]. For the 5-HT_1_A receptor, an association has been shown with increased proliferation of mitogen-activated B cells and improved resistance to apoptosis [28]. The evidence is based mainly on in vitro data and requires cautious extrapolation to physiological conditions.

Serotonin may participate in the humoral immune response through indirect mechanisms by affecting T-helper support of B cells, antigen presentation, and the cytokine microenvironment. Direct data showing marked enhancement of antibody production by 5-HT are less extensive than data on T-cell regulation. The role of serotonin in humoral responses remains plausible but not fully quantified [66].

Changes in serotonergic signaling are discussed in autoimmune diseases in which B-cell tolerance and autoantibody production are impaired. Direct causal links between 5-HT signaling in B cells and autoantibody formation remain supported by a limited number of studies [12,67].

### 3.3. Dendritic Cells as a Link Between Innate and Adaptive Immunity

Dendritic cells are professional antigen-presenting cells that initiate adaptive immune responses. They can take up serotonin through the SERT transporter and accumulate an intracellular 5-HT pool. This distinguishes dendritic cells from passive mediator-responsive cells and indicates an active role in serotonergic intercellular transfer.

After interaction with T cells, dendritic cells can release serotonin in the immunological synapse. In the cited study, this release enhanced T-cell activation and acted as an additional signal complementing classical antigen presentation [17].

In intestinal inflammation, serotonin increased the activation phenotype of dendritic cells, increased expression of co-stimulatory molecules, and changed cytokine production. Through these mechanisms, dendritic cells can direct T-cell differentiation toward different effector subtypes, while the direction of the response depends on the tissue context and inflammatory stimulus [26].

### 3.4. Serotonin and Immune Tolerance

Immune tolerance is maintained by the balance between effector and regulatory lymphocytes and by cytokines with anti-inflammatory properties. Serotonin can interfere with these processes through effects on Treg cells, macrophages, and dendritic cells [3,68]. In several models, 5-HT has been associated with increased IL-10 production and reduced excessive inflammatory responses [3].

Through 5-HT_7_ and 5-HT_2_B receptors, serotonin can enhance regulatory signals in the immune system, including anti-inflammatory transcriptional programs and production of mediators of inflammation resolution [3,23]. These effects have been shown mainly in macrophages and mixed immune models, but they may indirectly affect adaptive immunity [23].

In the macrophage studies cited above, these regulatory effects included reduced LPS-induced inflammatory mediators and a 5-HT7-PKA-associated increase in TGF-beta1 [19,36].

In chronic inflammation, impaired serotonergic regulation may be associated with loss of immune balance. In autoimmune diseases, changes in peripheral serotonin levels, receptor expression, and immune cell sensitivity to 5-HT have been described. The cause of these changes is not always established; some observations may reflect a secondary response to inflammation [12,68].

### 3.5. Serotonylation and Epigenetic Regulation

Serotonylation is the covalent attachment of serotonin to proteins through transglutaminases. It was first described for small GTPases and platelet activation, where serotonylation affected intracellular signaling and granule secretion [46]. Later, nuclear proteins were also identified as targets of serotonylation [47].

Histone serotonylation has been described for histone H3 and is associated with increased binding of the transcriptional complex TFIID to the H3K4me3 mark. This finding links serotonin to regulation of transcriptionally active chromatin and shows that 5-HT can act not only through membrane receptors but also through epigenetic mechanisms [47].

Biologically, this means that H3 serotonylation may function as a permissive chromatin mark that facilitates assembly of transcriptional complexes at active promoters. In immune cells, this concept is relevant because intracellular 5-HT may influence inflammatory gene expression through receptor-independent epigenetic mechanisms. However, direct evidence remains stronger in non-immune, tumor, and NETosis-related models than in defined T- or B-cell subsets; therefore, immune-cell-specific interpretation should remain cautious [21,47].

Direct data on T and B lymphocytes are currently less extensive than data from neuronal and tumor models [21,47]. The role of serotonylation in adaptive immunity remains insufficiently verified experimentally [21,46,47].

## 4. Clinical Role of Serotonin

**Figure 1:**
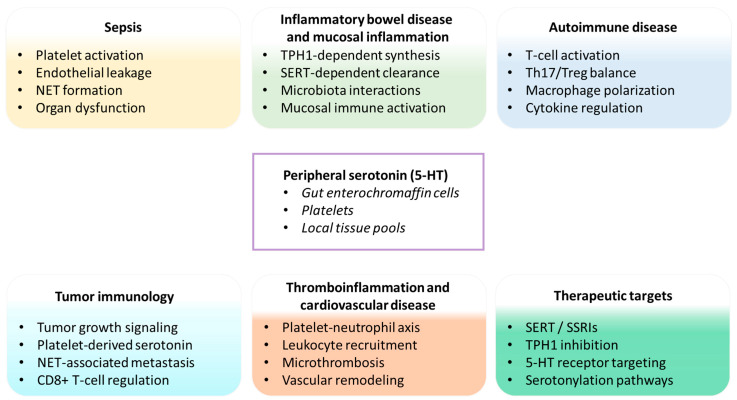
Clinical roles of peripheral serotonin.

Serotonin is involved in the regulation of several inflammatory and immune-mediated diseases, including sepsis, inflammatory bowel disease, autoimmune disorders, cardiovascular inflammation, thromboinflammation, and cancer-associated immune remodeling [10,19,52]. These associations reflect the ability of 5-HT to modulate platelet activation, endothelial permeability, leukocyte recruitment, macrophage polarization, T-cell responses, intestinal inflammation, and antitumor immunity. However, serotonin effects are receptor-, tissue-, and disease-stage-specific; therefore, 5-HT should be considered a context-dependent immunoregulatory mediator rather than a uniformly pro- or anti-inflammatory factor [32,37]. The main clinical contexts, associated immune mechanisms, and potential therapeutic targets of peripheral serotonin are summarized in Figure 1.

### 4.1. Acute Inflammation and Sepsis

In sepsis, the serotonergic system is associated with platelet activation, endothelial dysfunction, vascular hyperpermeability, neutrophilic inflammation, and microcirculatory impairment [3,8,10,69]. In septic shock, platelet activation and thrombocytopenia change serotonin availability; 5-HT measurements in whole blood, plasma, and platelets may therefore reflect different biological processes [3,70].

5-Hydroxyindoleacetic acid (5-HIAA) is the main serotonin metabolite and is considered a potential indicator of serotonin turnover in critical illness. In a clinical study of patients with septic shock, plasma 5-HIAA was associated with disease severity, duration of mechanical ventilation, length of intensive care unit stay, and adverse outcome. In the same study, serotonin increased endothelial cell permeability through a ROCK-dependent mechanism, suggesting a possible link between serotonin metabolism and vascular leakage in septic shock. The association does not prove that 5-HIAA is an independent causal target in sepsis, but it supports its interpretation as a clinical biochemical marker associated with vascular dysfunction [10].

Vascular permeability in sepsis is driven by endothelial barrier disruption, alterations in intercellular junctions, activation of RhoA/ROCK signaling, inflammatory cytokines, and damage to the glycocalyx. Serotonin can increase endothelial contractility and permeability in experimental models, indicating its involvement in vascular leakage [10,69]. Clinical interpretation of this pathway is limited because plasma serotonin concentration does not necessarily reflect local 5-HT concentration at platelet–endothelial contact sites [3,69].

NETosis contributes to antibacterial defense, but excessive formation of neutrophil extracellular traps in sepsis is associated with endothelial injury, microthrombosis, and organ dysfunction [58]. In an experimental model of sepsis-induced acute lung injury, platelet-derived 5-HT was associated with increased NET formation and lung tissue damage. These results are consistent with a model in which serotonin participates in sepsis pathogenesis through the platelet–neutrophil axis, but transferability of these findings to clinical sepsis in humans requires additional testing [19].

### 4.2. Autoimmune and Chronic Inflammatory Diseases

In autoimmune diseases, serotonin is considered a regulator of interactions among immune cells, the vascular wall, platelets, and the tissue microenvironment [12]. Its effects include regulation of T-cell activation, Th17 responses, macrophage polarization, cytokine production, and mucosal inflammation [4,12,27,34]. A consistent direction of these effects has not been established because serotonin can enhance or limit inflammation depending on receptor subtype, tissue, and disease stage [4,12].

In rheumatoid arthritis, the serotonergic system is associated with immune phenotype and bone-inflammatory processes [27,67]. In an experimental model of autoimmune arthritis, serotonin participated in inflammation through the Th17 response and bone resorption [27]. Genetic variation in a serotonin receptor gene was associated with changes in immune phenotype in rheumatoid arthritis [67]. In a 2025 clinical study, patients with rheumatoid arthritis, psoriatic arthritis, and axial spondyloarthritis had higher serotonin levels than healthy controls; however, this association should be treated as associative rather than causal [37].

In multiple sclerosis, serotonin is discussed in relation to regulation of T-cell responses, monocyte–macrophage activation, and neuroimmune interaction [29,50]. In natalizumab-treated patients with multiple sclerosis, 5-HT_7_ receptor expression on T lymphocytes was dysregulated, and receptor activation increased IL-10 production in PBMCs [29]. The result is consistent with a possible anti-inflammatory role of 5-HT_7_ signaling, but it cannot be generalized to all forms and stages of multiple sclerosis [29,50].

### 4.3. Serotonin and Intestinal Inflammation

The intestine is the main source of peripheral serotonin because mucosal enterochromaffin cells synthesize 5-HT through a TPH1-dependent pathway. Serotonin regulates intestinal motility, secretion, sensitivity, barrier functions, and mucosal immune responses [2,35]. In inflammatory bowel disease, the serotonergic system is associated with enterochromaffin cells, SERT-dependent 5-HT uptake, the microbiota, and local immune activation [34,35].

In the intestinal mucosa, enterochromaffin-cell-derived 5-HT is not only a source of circulating serotonin but also a local regulator of epithelial secretion, motility, barrier function, sensory signaling, and mucosal immune activation. During intestinal inflammation, changes in enterochromaffin-cell activity, TPH1-dependent synthesis, SERT-mediated clearance, and microbiota-derived tryptophan metabolites may alter local 5-HT availability. This is why gut serotonin should be treated as a local mucosal regulator [34,52].

Colitis models indicate that serotonin participates in the pathogenesis of intestinal inflammation. In mice with TPH1 deficiency or pharmacological reduction of peripheral serotonin synthesis, intestinal inflammation was less severe, indicating a contribution of enterochromaffin 5-HT to experimental colitis. The findings come from animal models and do not imply that serotonin blockade is universally effective in all forms of inflammatory bowel disease in humans [11].

SERT regulates extracellular 5-HT availability in the intestine by mediating serotonin reuptake and limiting its action in the mucosa. In a TNBS-induced colitis model, SERT deficiency aggravated inflammation, which is consistent with the view that impaired 5-HT clearance can support the inflammatory process. In inflammatory bowel disease, changes in SERT expression and serotonin metabolism have been described, but the causal link between these changes and disease activity remains incompletely defined [35,36].

The intestinal data provide a useful example of apparently divergent effects of serotonin. Reduction of TPH1-dependent 5-HT synthesis decreased the severity of experimental colitis, whereas loss of SERT-dependent 5-HT clearance aggravated TNBS-induced colitis [11,36]. These results are mechanistically compatible: TPH1 deficiency reduces 5-HT production, whereas SERT deficiency prolongs extracellular 5-HT exposure by impairing reuptake.

The intestinal microbiota can influence the serotonergic system through metabolites that affect enterochromaffin cells and tryptophan metabolism [35]. Serotonin, in turn, can modify the mucosal immune microenvironment, including dendritic cells, macrophages, and T-cell responses. In intestinal inflammation, serotonin is part of the microbiota–epithelium–immune cell axis rather than an isolated inflammatory mediator [34,35].

### 4.4. Serotonin and Tumor Immunology

In the tumor microenvironment, the serotonergic system can affect tumor growth, inflammation, angiogenesis, platelet–tumor interactions, and cytotoxic T-cell function. The direction of 5-HT effects depends on tumor type, receptor profile, local serotonin metabolism, platelet activity, and the composition of the immune infiltrate [13,32,71].

In hepatocellular carcinoma, serotonin has been studied as a factor that may support tumor growth. In Huh7 and HepG2 cell lines, serotonin stimulated tumor cell growth and survival under nutrient deprivation, and this effect was linked to activation of downstream mTOR targets [38]. Clinical data on intraplatelet serotonin also indicate an association between the platelet 5-HT pool and HCC prognosis: post-resection depletion of intraplatelet serotonin was considered a possible marker of early recurrence, and higher intraplatelet 5-HT combined with YAP expression was associated with poor prognosis [39,40].

In breast cancer, changes in the local serotonergic system have been described, including expression of components involved in 5-HT synthesis, transport, and receptor signaling in tumor cells. In the study by Pai et al., altered serotonin physiology in human breast tumors was associated with increased growth and survival of tumor cells [41]. In triple-negative breast cancer, TPH1 and the 5-HT_7_ receptor were preferentially expressed in tumor tissue and supported progression through autocrine serotonin signaling [42].

Data on triple-negative breast cancer show that the 5-HT_7_ receptor may have prognostic value and contribute to tumor cell proliferation through a FOXM1-dependent pathway [43]. In models of breast tumor-initiating cells, serotonergic system antagonists reduced the frequency of tumor-initiating cells and synergized with chemotherapy in reducing xenograft size, indicating pharmacological sensitivity of serotonergic pathways in selected breast cancer models [44].

Macrophages in the tumor microenvironment may respond to serotonin by changing their transcriptional profile, including anti-inflammatory and profibrotic programs. This phenotype can support tissue remodeling and an immunosuppressive microenvironment, but it is not a universal feature of all tumors [23,32]. Neutrophils and NETs may also participate in tumor progression and metastasis [20]. Liu et al. described a link between 5-HT, histone serotonylation, histone citrullination, NETs, and liver metastasis; the proposed pathway requires independent confirmation in different tumor models [21].

SERT has been proposed as a possible immune checkpoint for CD8+ T cells in the tumor microenvironment. Li et al. showed that SERT suppresses the CD8+ T-cell antitumor response by regulating the intratumoral serotonin axis, whereas inhibition of SERT with selective serotonin reuptake inhibitors increased antitumor activity and synergized with anti-PD-1 therapy in experimental models [13,45].

These findings reflect cell-compartment-specific effects rather than a single tumor-wide action of 5-HT: serotonergic signaling can support tumor-cell growth [38,42], whereas SERT inhibition in CD8+ T cells enhances antitumor responses and PD-1 blockade in experimental models [13,45].

### 4.5. Serotonin, Thromboinflammation, and Cardiovascular Disease

Platelets are the main circulating reservoir of peripheral serotonin and release 5-HT upon activation. In the vascular system, serotonin affects vascular tone, platelet function, smooth muscle cells, endothelial permeability, and inflammatory leukocyte recruitment [3,8,69]. These properties link serotonin to thromboinflammation, in which hemostasis, innate immunity, and vascular inflammation form a single pathological circuit [8,58].

Platelet serotonin increases neutrophil recruitment to sites of acute inflammation in mice. The finding links platelet activation with leukocyte inflammation and endothelial dysfunction. NETs can promote thrombosis through extracellular DNA, histones, and neutrophil granule proteins that interact with platelets and coagulation factors [8,58].

In the vascular wall, serotonin can stimulate vascular smooth muscle cell proliferation and contribute to vascular remodeling. In early experimental studies, 5-HT stimulated vascular smooth muscle cell proliferation, and the combined action of serotonin and thromboxane A2 enhanced this effect. The findings are relevant to vascular remodeling but should be separated from clinically proven cardiovascular risk factors [33].

### 4.6. Pharmacological Modulation of the Serotonergic System

Pharmacological intervention in the serotonergic system includes selective serotonin reuptake inhibitors, antagonists and agonists of specific 5-HT receptors, TPH1 inhibitors, and drugs that affect 5-HT metabolism. SSRIs block SERT and change the distribution of serotonin between cellular and extracellular compartments; in an immunological context, this can affect platelets, T cells, dendritic cells, macrophages, and the tumor microenvironment [3,61,69].

In sepsis, experimental data indicate possible immunometabolic effects of fluoxetine. Gallant et al. showed that fluoxetine enhanced IL-10-dependent metabolic defense mechanisms in an experimental sepsis model. This result does not support clinical use of fluoxetine as standard sepsis therapy because controlled clinical trials are needed to assess safety, dosing, and effects on outcomes [14].

In tumor immunology, SERT inhibition is considered a way to enhance CD8+ T-cell antitumor responses. In tumor models, SSRIs increased the intratumoral serotonin signal and enhanced the effect of PD-1 blockade; this supports the interpretation of SERT as a target for combined immunotherapy [13,45].

This concept differs from the direct cytotoxic use of serotonergic drugs against tumor cells. In breast cancer models, antagonists of serotonergic pathways and drugs affecting SERT reduced the activity of tumor-initiating cells and enhanced the effect of chemotherapy, but these results were obtained mainly in preclinical systems and do not define a standard of clinical treatment [42,43,44].

TPH1 inhibitors represent a separate approach to reducing peripheral serotonin synthesis without directly suppressing central serotonergic transmission. In experimental colitis, reduction of TPH1-dependent serotonin synthesis reduced inflammation severity. Use of this approach in inflammatory diseases requires consideration of physiological intestinal 5-HT functions, including motility, secretion, and interaction with the microbiota [2,11,35].

### 4.7. Limitations of Clinical Interpretation

Interpretation of circulating 5-HT requires explicit identification of the sample matrix. Whole-blood 5-HT largely represents the platelet pool and is influenced by platelet count and storage capacity. Platelet-poor plasma more closely reflects the freely circulating fraction, but even minor platelet contamination or ex vivo activation can markedly increase measured concentrations. Serum values are higher because clotting releases platelet 5-HT, whereas tissue concentrations reflect local synthesis, uptake, metabolism, and release [3,7,70].

Reported 5-HT concentrations differ markedly depending on the biological matrix. Serum and whole-blood values often fall in the tens-to-hundreds ng/mL range because they largely reflect platelet-associated serotonin, whereas carefully prepared platelet-poor plasma contains a much smaller free circulating fraction and may show low ng/mL or low nmol/L values [37,72,73]. These numbers should not be interpreted as universal biological cutoffs because they depend on sample type, platelet contamination, collection and centrifugation protocol, storage conditions, analytical platform, and laboratory-specific reference intervals.

Central and peripheral serotonin systems are partly separate. Peripheral 5-HT is synthesized mainly in the intestine through TPH1, whereas central serotonin is synthesized in neurons through TPH2. Because serotonin poorly crosses the blood–brain barrier, peripheral 5-HT concentrations are not a direct indicator of central serotonergic activity. Clinical studies of inflammation, psychoneuroimmunology, and depression should not mechanically transfer peripheral 5-HT data to central mechanisms [2].

Species differences limit the transfer of data from mouse models to humans. Receptor expression, platelet biology, tryptophan metabolism, microbiota composition, and immune responses differ between species [35,45]. Results from models of colitis, sepsis, arthritis, and tumor growth should be confirmed in human cells, biological samples, and clinical cohorts [10,11,13,27].

Serotonin effects are dose-dependent and receptor-specific. The same mediator can produce different effects through 5-HT_1_, 5-HT_2_, 5-HT_3_, 5-HT_4_, and 5-HT_7_ receptors. Differences between acute and chronic inflammation also change interpretation: in acute inflammation, serotonin may participate in leukocyte recruitment and vascular permeability, whereas in chronic inflammation it may contribute to tissue remodeling, immune regulation, and fibrosis [8,23,45]. Serotonin should not be classified as an exclusively pro-inflammatory or anti-inflammatory mediator.

## 5. Conclusions

The reviewed data show that serotonin has regulatory functions in innate immunity, adaptive responses, and clinical inflammatory phenotypes. Peripheral 5-HT participates in leukocyte recruitment, platelet–immune interactions, regulation of macrophage phenotype, dendritic cell activity, T- and B-lymphocyte responses, vascular permeability, and tissue remodeling. These effects depend on cellular context, local mediator concentration, receptor expression, and disease stage.

Data on innate immunity link serotonin with early inflammatory phases, immunothrombosis, and the neutrophil–platelet axis. In adaptive immunity, 5-HT affects T-cell activation, intercellular communication through antigen-presenting cells, and, probably, mechanisms of immune tolerance. Clinical observations link serotonergic dysregulation with sepsis, inflammatory bowel disease, autoimmune pathology, cardiovascular inflammation, and the tumor microenvironment.

Interpretation is limited by differences between plasma, serum, and platelet serotonin, species differences in experimental models, and variable consideration of receptor specificity. In addition, serotonin cannot be assigned exclusively to pro-inflammatory or anti-inflammatory mediators because the direction of its action is determined by biological context.

Further tasks include standardizing clinical measurement of serotonin and its metabolites, mapping 5-HT receptor expression in human immune subpopulations, studying serotonylation as a mechanism of immune regulation, and assessing pharmacological modulation of SERT, TPH1, and specific 5-HT receptors as therapeutic approaches. Addressing these tasks is necessary to assess the applicability of serotonergic targets in sepsis, autoimmune diseases, chronic inflammation, and tumor immunopathology.

## Figures and Tables

**Table 1:** Serotonergic components reported in immune and inflammation-associated cell/tissue types.

Cell/Tissue Type	Serotonergic Components Reported	Evidence/Functional Readout	Main Implication	Key References
Platelets	SERT/SLC6A4, VMAT2, dense-granule 5-HT	5-HT uptake, storage, and release during platelet activation	Main circulating reservoir of peripheral 5-HT; link between hemostasis, leukocyte recruitment, and vascular inflammation	[3,7,8]
Neutrophils	SERT-dependent 5-HT uptake; TGM2/PAD4-linked serotonylation/citrullination pathway in NETosis models	Neutrophil recruitment, platelet–neutrophil interaction, NET formation	Connection between serotonin, immunothrombosis, sepsis-associated lung injury, and tumor-associated NETosis	[8,19,20,21]
Monocytes/macrophages	5-HT_2_B, 5-HT_7_, and 5-HT_2_C receptors	Cytokine and chemokine release, transcriptional profile changes, polarization, phagocytic and migratory activity	Context-dependent anti-inflammatory, immune-regulatory, chemotactic, and profibrotic effects	[22,23,24,25]
Dendritic cells	SERT/SLC6A4; intracellular 5-HT pool	5-HT uptake and release at the dendritic cell-T-cell immunological synapse; activation phenotype and cytokine changes in intestinal inflammation	Serotonergic link between innate sensing, antigen presentation, and T-cell activation	[17,26]
T lymphocytes	5-HT_7_ receptor; SERT/SLC6A4; de novo 5-HT synthesis reported in activated cells	Early T-cell activation, proliferation, migration, cAMP-dependent signaling, cytoskeletal changes	Co-stimulatory and regulatory effects depending on activation state, receptor expression, and inflammatory context	[9,27]
B lymphocytes	5-HT1A receptor and other serotonergic components	Mitogen-activated survival and proliferation; resistance to apoptosis	Possible modulation of humoral responses, although direct evidence remains less extensive than for T cells	[28,29]
Mast cells	Stored and released 5-HT	Granule mediator release after activation	Local regulation of vascular permeability, smooth muscle activity, and neuroimmune signaling	[15,30,31]
Eosinophils/allergic airway inflammation	5-HT2 receptor-dependent signaling in experimental systems	DOI-sensitive modulation of airway hyperresponsiveness, mucus production, inflammatory infiltration, and eosinophil recruitment	Example of anti-inflammatory 5-HT_2_-dependent effects in selected allergic inflammation models	[16]
Endothelium/vascular wall	5-HT-responsive permeability and contractility pathways; ROCK-related endothelial permeability response	Endothelial barrier regulation, vascular leakage, smooth muscle cell proliferation	Vascular permeability, thromboinflammation, and vascular remodeling	[10,32,33]
Intestinal mucosa/Inflammatory bowel disease context	Enterochromaffin-cell TPH1-dependent 5-HT synthesis; SERT-mediated 5-HT clearance; microbiota-related tryptophan metabolism	Reduced colitis after TPH1 deficiency or inhibition; aggravated colitis after SERT deficiency; microbiota–epithelium–immune cell interactions	Local mucosal 5-HT availability depends on synthesis, clearance, microbiota, and tissue exposure time	[2,11,34,35,36]
Tumor microenvironment	SERT, TPH1, 5-HT7 receptor, platelet-derived 5-HT	Tumor-cell growth, CD8+ T-cell antitumor response, platelet–tumor interactions, NET-associated metastasis	Serotonin may support tumor-cell programs in some compartments, whereas SERT inhibition may enhance antitumor immunity in others	[13,21,37,38,39,40,41,42,43,44,45]

**Table 2:** Functional evidence and downstream signaling of 5-HT receptors and related serotonergic mechanisms in immune and inflammation-associated contexts.

Receptor/Serotonergic Mechanism	Main Signaling or Mechanism	Main Cell/Tissue Context	Functional Evidence Described in The Review	Interpretation for Context-Dependent or Opposing Effects	Key References
5-HT_1_A receptor	Mainly Gi/o-coupled signaling; generally associated with reduced cAMP	B lymphocytes and mitogen-activated lymphocytes	Increased survival and proliferation of mitogen-activated B cells; improved resistance to apoptosis	May support lymphocyte expansion in selected in vitro settings; physiological relevance requires cautious interpretation	[47,48]
5-HT_2_B receptor	Gq/11-PLC-IP3/Ca^2+^-linked signaling; AhR-related transcriptional regulation	Human monocyte-derived macrophages	M2-like macrophage polarization; altered expression of immune-regulatory and xenobiotic-response genes	Can reduce inflammatory mediator production while also supporting tissue-remodeling or regulatory programs	[19,37]
5-HT_2_C receptor	Gq/11-linked Ca^2+^ signaling	Murine alveolar macrophages	Increased intracellular Ca^2+^ and enhanced CCL2/MCP-1 production	May promote chemokine-dependent leukocyte recruitment in lung immune responses	[38]
5-HT_2_ receptor-dependent pathway	Gq/11-Ca^2+^-associated signaling; DOI-sensitive pathway in experimental airway inflammation	Allergic airway inflammation/eosinophil-related context	DOI reduced airway hyperresponsiveness, mucus production, inflammatory infiltration, and eosinophil recruitment in a mouse asthma model	Demonstrates that 5-HT2-related signaling may be anti-inflammatory in some models despite pro-inflammatory effects in other settings	[16]
5-HT_3_ receptor	Ligand-gated cation channel; ionotropic signaling	Immune-cell context less defined in the current review	Mentioned as the only ionotropic 5-HT receptor family, in contrast to GPCR-type 5-HT receptors	Important for receptor classification and mechanistic diversity, but immune-specific functional evidence is less developed	[18]
5-HT_7_ receptor	Gs-cAMP-PKA signaling	T lymphocytes, macrophages, PBMCs, inflammatory disease contexts	T-cell co-stimulation, cytoskeletal changes, migration, early activation; macrophage anti-inflammatory/profibrotic transcriptional profile; IL-10-associated effects in PBMCs	Can limit inflammatory mediator production but also support profibrotic tissue remodeling, explaining apparently divergent outcomes	[9,36,49]
SERT/SLC6A4	Cellular uptake of extracellular 5-HT; regulation of extracellular and intracellular 5-HT availability	Platelets, dendritic cells, neutrophils, CD8+ T cells, tumor microenvironment	Platelet 5-HT storage; dendritic cell-to-T-cell serotonergic transfer; SERT-dependent neutrophil 5-HT uptake during NETosis; regulation of CD8+ T-cell antitumor activity	Determines whether 5-HT acts extracellularly through receptors or intracellularly through uptake-dependent mechanisms; effects depend strongly on cell compartment	[3,7,13,17,33,50]
TPH1-dependent peripheral 5-HT synthesis	Peripheral serotonin synthesis, especially in enterochromaffin cells	Intestinal mucosa, inflammatory bowel disease, tumor-related contexts	TPH1 deficiency or pharmacological reduction of peripheral 5-HT synthesis reduced experimental colitis; TPH1/5-HT7 autocrine signaling supported progression in triple-negative breast cancer	Reduced synthesis and impaired clearance can produce different inflammatory outcomes; local 5-HT production is tissue- and disease-dependent	[2,11,51,52]
VMAT2-dependent vesicular storage	Vesicular accumulation of 5-HT in dense granules	Platelets	Platelets take up serotonin through SERT and store it in dense granules through VMAT2 before release during activation	Explains why whole-blood and serum 5-HT largely reflect the platelet-associated serotonin pool rather than free circulating 5-HT	[3,7]
TGM2/PAD4-linked serotonylation/citrullination	Receptor-independent intracellular mechanism; H3Q5ser/H3cit modification	Neutrophils/NETosis-related models	SERT-dependent 5-HT uptake promoted TGM2-dependent histone serotonylation coupled with PAD4-dependent citrullination; this facilitated chromatin decondensation and NET formation	Shows that 5-HT can regulate immune responses through intracellular epigenetic/post-translational mechanisms, not only membrane receptors	[22,23,33]
ROCK-related endothelial response	Endothelial contractility and permeability pathway	Endothelium/septic shock context	5-HT increased endothelial permeability through a ROCK-dependent mechanism in the septic shock-related study	Connects serotonergic signaling to vascular leakage, but plasma 5-HT does not necessarily reflect local platelet–endothelial 5-HT exposure	[10,53]

## Data Availability

No new data were created or analyzed in this study.

## Abbreviations

The following abbreviations are used in this manuscript:

5-HT	5-hydroxytryptamine
5-HIAA	5-hydroxyindoleacetic acid
5-HT_1_–5-HT_7_	Serotonin receptor families 1–7
HTR1-HTR7	Genes encoding serotonin receptor families 1-7
AhR	Aryl hydrocarbon receptor
anti-PD-1	Anti-programmed cell death protein 1
Ca^2+^	Calcium ion
CCL2	C-C motif chemokine ligand 2
CD8+ T cells	CD8-positive T lymphocytes
DOI	2,5-dimethoxy-4-iodoamphetamine
DNA	Deoxyribonucleic acid
FOXM1	Forkhead box M1
H3cit	Citrullinated histone H3
H3K4me3	Trimethylation of lysine 4 on histone H3
H3Q5ser	Histone H3 glutamine 5 serotonylation
HCC	Hepatocellular carcinoma
HepG2	Human hepatoblastoma-derived cell line
Huh7	Human hepatocellular carcinoma cell line
IBD	Inflammatory bowel disease
IL-1β	Interleukin-1 beta
IL-6	Interleukin-6
IL-10	Interleukin-10
LPS	Lipopolysaccharide
M0	Non-polarized macrophage state
M1	Classically activated macrophage phenotype
M2	Alternatively activated macrophage phenotype
MCP-1	Monocyte chemoattractant protein-1
mTOR	Mechanistic target of rapamycin
NETs	Neutrophil extracellular traps
NETosis	Neutrophil extracellular trap formation
PAD4	Peptidyl arginine deiminase 4
PBMCs	Peripheral blood mononuclear cells
PD-1	Programmed cell death protein 1
PKA	Protein kinase A
ROCK	Rho-associated coiled-coil-containing protein kinase
SERT	Serotonin transporter
SLC6A4	Solute carrier family 6 member 4
SSRI	Selective serotonin reuptake inhibitor
T cells	T lymphocytes
B cells	B lymphocytes
TGF-β1	Transforming growth factor beta 1
TFIID	Transcription factor IID
Th1	T helper 1 cells
Th2	T helper 2 cells
Th17	T helper 17 cells
TNBS	2,4,6-trinitrobenzene sulfonic acid
TNF-α	Tumor necrosis factor alpha
TPH1	Tryptophan hydroxylase 1
TPH2	Tryptophan hydroxylase 2
Treg	Regulatory T cells
VMAT2	Vesicular monoamine transporter 2
YAP	Yes-associated protein
